# Longevity of Physicians

**Published:** 1873-08

**Authors:** 


### LONGEVITY OF PHYSICIANS.

DOCTORS do not live as long as either the lawyer or the clergyman ; the anxieties and unavoidable exposures of the earlier years of practice make sad inroads on the health, and many are cut off prematurely ; but if they survive forty-five, their chances for four score are very good, because by that time they have learned, by experience, the importance of taking care of their health, and they know better how to do it; then, again, they have better learned the ways of disease ; they can look at it with less indecision, and escape that painful "balancing," which is a great draught on the nervous system. At forty-five the physician finds himself more at leisure, is better able to consult his comfort, and can make his patients wait his convenience ; he is more independent, takes the loss of a patient less to heart, looking upon it more as an inevitable event, he having done all he could, and there his duty ends.

Of the physicians dying in 1872 fourteen averaged eighty-five years, but in all probability four times as many died before forty-five.

